# Training Health Workers

**Published:** 1920-02-14

**Authors:** 


### Training Health Workers.

THE Royal Sanitary Institute has a good syllabus of lectures and demonstrations for the spring term starting the first week in February. There is a sanitary officers' course covering a wide range of subjects, including those scheduled for the examinations of the Institute and Sanitary Inspectors' Examination Board; there is a course of practical training for candidates as meat inspectors, and an interesting and comprehensive programme of a valuable character for those desirous of qualifying as women health visitors and child-welfare workers.

